# Implementation of an Early Transition to Oral Antibiotics for Patients With Nonstaphylococcal Bacteremia via Real-Time Stewardship Intervention

**DOI:** 10.1093/ofid/ofag120

**Published:** 2026-03-04

**Authors:** Tyler Tate, Brandon J Smith, J Alex Viehman, Ryan K Shields

**Affiliations:** Division of Infectious Diseases, University of Pittsburgh School of Medicine, Pittsburgh, Pennsylvania, USA; Antibiotic Management Program, University of Pittsburgh Medical Center, Pittsburgh, Pennsylvania, USA; Division of Infectious Diseases, University of Pittsburgh School of Medicine, Pittsburgh, Pennsylvania, USA; Antibiotic Management Program, University of Pittsburgh Medical Center, Pittsburgh, Pennsylvania, USA; Division of Infectious Diseases, University of Pittsburgh School of Medicine, Pittsburgh, Pennsylvania, USA; Antibiotic Management Program, University of Pittsburgh Medical Center, Pittsburgh, Pennsylvania, USA; Division of Infectious Diseases, University of Pittsburgh School of Medicine, Pittsburgh, Pennsylvania, USA; Antibiotic Management Program, University of Pittsburgh Medical Center, Pittsburgh, Pennsylvania, USA; Center for Innovative Antimicrobial Therapy, University of Pittsburgh School of Medicine, Pittsburgh, Pennsylvania, USA

**Keywords:** bacteremia, oral antibiotics, stewardship

## Abstract

**Background:**

Oral antibiotics are effective in treating uncomplicated bloodstream infections (BSIs), but they are underused. The objective of this study was to evaluate the impact of a standardized antimicrobial stewardship intervention for early transition to oral antibiotics (“oral transition”) for patients with uncomplicated BSIs.

**Methods:**

A quasi-experimental study was conducted before and after implementation of a standardized stewardship intervention for patients with uncomplicated non-staphylococcal BSI over a 4-month period. Rates of oral transition and clinical outcomes were compared before and after intervention. The primary outcome was the rate of oral transition. Clinical failure (relapsed bacteremia, infection-attributable death, or new deep-seated infection with the same BSI organism) was a secondary outcome.

**Results:**

A total of 187 and 177 BSIs were evaluated before and after the intervention, respectively. Overall, 44% (82 of 187) and 43% (76 of 177) met pre-specified criteria for oral transition. Overall oral transition rates increased from 59% (48 of 82) before to 93% (71 of 76) after intervention (*P* < .01). Baseline characteristics, including severity of illness, underlying diseases, and the BSI source, were similar between groups. Clinical failure occurred within 90 days in 7% (6 of 82) and 7% (5 of 76) of patients in the pre- and post-intervention groups, respectively. The total mean antibiotic duration was shorter in the post-intervention group (10.76 vs 12.15 in the pre-intervention group; *P* = .02). Secondary outcomes, including length of stay, 30-day readmission rate, and antibiotic adverse events, were similar between groups.

**Conclusions:**

The implementation of a real-time antimicrobial stewardship intervention resulted in an increased rate of transition to oral antibiotics and fewer overall days of therapy, without an observed difference in clinical or safety outcomes.

Bacterial bloodstream infections (BSI) are common [1] and are associated with significant morbidity and mortality [2]. Choosing an optimized antibiotic treatment regimen is critical to assure clinical cure. Intravenous antibiotics have been historically perceived as the standard of care for serious bacterial infections, including BSIs [3]; however, the necessity of intravenous treatment is unclear. This is in part due to the lack of evidence supporting greater efficacy of intravenous therapy compared with appropriately dosed, highly bioavailable oral antibiotics and an increased rate of adverse events associated with intravenous therapy [3, 4]. Moreover, outpatient intravenous antimicrobial therapy has been associated with increased caregiver burden, healthcare costs, missed work and lower quality of life [5], providing added incentives to transition patients to oral antibiotics before hospital discharge.

Recently, the BALANCE trial [6] demonstrated that 7 days of antibiotic therapy was noninferior to 14 days of therapy in patients with BSI. Overall, 60.8% of patients received oral antibiotic treatment. These data add to several randomized controlled trials that have now concluded that oral antibiotics are noninferior to intravenous antibiotics for the treatment of uncomplicated BSI [7, 8]. To translate these data to clinical practice, real-world implementation studies are needed to define stewardship strategies that accurately and rapidly identify patients who stand to benefit from early transition to oral therapy (“oral transition”). We hypothesized that a standardized stewardship intervention would improve rates of oral transition among patients with uncomplicated nonstaphylococcal BSI at our center and that the outcomes of these patients would be similar to those treated with intravenous therapy.

## METHODS

We conducted a quasi-experimental study comparing a retrospective cohort and a prospectively evaluated intervention cohort of patients with uncomplicated nonstaphylococcal BSIs who received a real-time stewardship intervention. In brief, consecutive cases of BSIs due to *Enterobacterales*, *Streptococcus* spp, and *Enterococcus faecalis* were reviewed daily by our stewardship team, which included infectious diseases (ID)–trained physicians and pharmacists, through real-time electronic alerts received at the time of Gram-stain results. After pathogen identification by rapid molecular testing of positive blood cultures, the microbiology laboratory directly communicated results to the stewardship provider, who maintained the list of patients with positive blood cultures daily.

Next, patients were screened for pre-specified criteria for the transition to oral antibiotics, from 1 August to 30 November 2024 (Supplementary Table 1). Once oral transition criteria were met, the primary team received real-time stewardship recommendations for a preferred oral antibiotic regimen (Supplementary Table 2), which included patients able to take medications orally or through an enteral feeding tube. To measure the impact of real-time intervention, we compared prospectively evaluated patients with a retrospective cohort of consecutive cases of BSIs from 1 August to 30 November 2022. During this period, cases were managed according to the standard of care through audit and feedback practices by daily review of BSIs by a stewardship provider, but without the specific real-time guidance and intervention for early oral transition.

The primary outcome of the study was the rate of oral transition among eligible patients. Secondary outcomes included clinical and safety measures. A composite end point for clinical failure within 30 or 90 days from index positive blood culture was defined as relapsed bacteremia with the same species, infection-attributable death, or new deep-seated infection due to the index BSI pathogen. Demographic and baseline characteristics were collected, as well as days of intravenous therapy received, total antibiotic treatment duration, the rate of oral transition before discharge, length of stay, the 30-day readmission rate, adverse antibiotic events, 30- and 90-day mortality rates, and the rate of discharge to home.

Baseline characteristics and outcomes for the pre- and post-intervention groups were compared by means of Wilcoxon rank sum tests for continuous variables and χ² or Fisher exact tests for categorical variables. To determine the impact of real-time intervention on oral transition, a multivariable logistic regression model was constructed to identify factors association with successful oral transition, using variables with a *P* value <.10 on univariate analysis. Results are reported as adjusted odds ratios with 95% confidence intervals. Multicollinearity was evaluated using variance inflation factors. All tests were 2 sided, with differences considered statistically significant at *P* < .05.

All data for this study were collected with a waiver of informed consent granted by the University of Pittsburgh Institutional Review Board (STUDY 22070065).

## RESULTS

A total of 187 BSIs were reviewed during the pre-intervention period and 177 in the post-intervention period. The corresponding proportions of patients who met criteria for oral transition were 44% (82 of 187) and 43% (76 of 177), respectively. Severity of illness, as measured by baseline Pitt bacteremia score (at the time of first positive blood culture), was similar between groups (mean [SD] score, 2.99 [2.5] in the pre-intervention vs 2.25 [2.0] in the post-intervention group; *P* = .24). Baseline characteristics were similar between groups, as were rates of source control intervention (Table 1). Differences were observed in the median age (68 vs 62 years; *P* = .02), the presence of cardiac devices (15% in the preintervention vs 4% in the postintervention group; *P* = .03), and the rate of ID consultation (51% vs 67%; *P* = .05). The most common sources of bacteremia were similar between groups and most commonly included genitourinary (38% in the pre-intervention vs 29% in the post-intervention group; *P* = .31) or intra-abdominal (27% vs 28%; *P* > .99) infections (Table 1). Gram-negative pathogens were cultured in 79% of patients in the pre-intervention and 72% in the post-intervention group (Supplementary Table 3).

**Table 1.** Baseline Demographic and Clinical Characteristics and Sources of Bloodstream Infections

Characteristic or Infection Source	Patients, No. (%)^a^		*P* Value
	Before Intervention (n = 82)	After Intervention (n = 76)	
Age, median (IQR), y	68 (56–75)	62 (51–73)	.02
Male sex	50 (61)	42 (55)	.52
Charlson comorbidity index, mean (SD)	5.93 (3.2)	5.41 (3.7)	.30
Pitt bacteremia score, mean (SD)	2.99 (2.8)	2.25 (2.0)	.24
Cardiac device	12 (15)	3 (4)	.03
PICC or vascular device	15 (18)	10 (13)	.39
Past or current IVDU	8 (10)	7 (9)	>.99
Transplant recipient	7 (9)	5 (7)	.77
ID consultation	42 (51)	51 (67)	.05
Source control intervention	36 (44)	31 (41)	.87
Repeat blood culture obtained	67 (82)	55 (72)	.16
Infection source			
GU	31 (38)	22 (29)	.31
GI/intra-abdominal	22 (27)	21 (28)	>.99
Unknown or >1 possibility	19 (23)	12 (16)	.32
SSTI	5 (6)	12 (16)	.07
Catheter associated	4 (5)	8 (11)	.23
IVDU	1 (1)	0 (0)	>.99
Pulmonary	0 (0)	1 (1)	.48

Abbreviations: GI, gastrointestinal; GU, genitourinary; ID, infectious diseases; IQR, interquartile range; IVDU, intravenous drug use; PICC, peripherally inserted central catheter; SSTI, skin and soft-tissue infection.

^a^Data represent no. (%) of patients unless otherwise specified.

The rate of oral antibiotic transition in patients who met criteria significantly increased from 59% (48 of 82) before to 93% (71 of 76) after intervention (*P* < .01) (Table 2). Across all patients eligible for oral antibiotics, 75% underwent the transition. In multivariable logistic regression, ICU admission was independently associated with lower odds of changing to oral antibiotics (adjusted odds ratio, 0.10 [95% confidence interval, .03–.34]), whereas patients in the post-intervention group (17.23 [4.60–64.50]) and those with a urinary tract infection source (4.23 [1.16–15.47]) were associated with higher odds of oral transition (Supplementary Table 4). The oral antibiotics used can be found in the Supplementary materials (Supplementary Table 5 and Supplementary Figure 1). Clinical failure occurred within 90 days in 7% (6 of 82) and 7% (5 of 76) of patients in the pre- and post-intervention groups, respectively. There was no significant difference in 30-day mortality rates (9% vs 3%; *P* = .17); however, fewer days of intravenous therapy were prescribed in the post-intervention group (median [interquartile range], 6.4 [3.3–10.8] days in the pre-intervention vs 3.9 [2.5–6.8] in the post-intervention group; *P* < .01). Among patients transitioned to oral antibiotics, more in the post-intervention group received the first dose of oral antibiotic therapy before discharge (46% [22 of 48] in the preintervention vs 79% [56 of 71] in the post-intervention group; *P* < .01). The total antibiotic therapy duration was shorter in the post-intervention group (mean [SD], 12.15 [4.0] days in the pre-intervention vs 10.76 [3.2] days in the post-intervention group; *P* = .02).

**Table 2.** Clinical Outcomes of Patients With Uncomplicated Bloodstream Infections Before and After Stewardship Intervention

Outcome	Patients, No. (%)^a^		*P* Value
	Before Intervention (n = 82)	After Intervention (n = 76)	
LOS after index blood culture, median (IQR), d	7.4 (3.5–14.5)	6.6 (3.5–12.5)	.51
Antibiotic duration, mean (SD), d	12.15 (4.0)	10.76 (3.2)	.02
Composite clinical failure at 30 d	4 (5%)	3 (4%)	>.99
Composite clinical failure at 90 d	6 (7)	5 (7)	>.99
*Clostridioides difficile* infection	1 (1)	1 (1)	>.99
New deep-seated infection	4 (5)	2 (3)	.68
Readmission (at 30 d)	19 (23)	22 (29)	.47
Relapsed bacteremia (at 30 d)^b^	2 (2)	1 (1)	>.99
Mortality rate (at 30 d)	7 (9)	2 (3)	.17
Antibiotic adverse events^c^	5 (6)	3 (4)	.72
Discharge to home	50 (61)	54 (71)	.24
Total eligible for oral antibiotics	82 (44)	76 (43)	.83
Completed transition to oral antibiotics	48 (59)	71 (93)	<.01
Duration of intravenous therapy, median (IQR), d	6.4 (3.3–10.8)	3.9 (2.5–6.8)	<.01
Transition to oral therapy before discharge	22 (46)	56 (79)	<.01

Abbreviations: IQR, interquartile range; LOS, length of stay.

^a^Data represent no. (%) of patients unless otherwise specified.

^b^Relapsed bacteremia was defined as a positive blood culture with the same species within 30 days after the index blood culture.

^c^Antibiotic adverse events were defined as any unintended and harmful effects occurring in association with the administration of an antibiotic, where a causal relationship is at least reasonably possible.

Length of stay after the index positive blood culture (median [interquartile range], 7.4 [3.5–14.5] days in the pre-intervention vs 6.6 [3.5–12.5] days the post-intervention group; *P* = .51), antibiotic adverse events (6% vs 4%; *P* = .72), rate of discharge home (61% vs 71%; *P* = .24), *Clostridioides difficile* infection (1% vs 1%; *P* > .99), and the rate of re-admission at 30 days (23% vs 29%; *P* = .47) did not differ between groups (Table 2).

## DISCUSSION

In the current study, we successfully implemented standardized criteria to transition patients with uncomplicated non-staphylococcal BSI to oral antibiotics as soon as clinical stability criteria were met. This approach resulted in a 93% success rate, fewer days of intravenous antibiotics, and shorter mean durations of therapy. On average, patients were transitioned to oral antibiotics within 4 days, which did not negatively affect longer-term outcomes at 90 days. These findings are consistent with prior literature demonstrating that audit and feedback is among the most effective types of stewardship intervention [9]. Our data further attest to the importance of antibiotic stewardship teams, who are uniquely positioned to review blood cultures and intervene early in the treatment course in an effort to optimize antibiotic selection, duration, and use. Moreover, the implementation of this intervention did not significantly increase workload and was integrated seamlessly into the current workflow.

Total antibiotic treatment durations were determined by each patient's provider, with guidance from the antibiotic stewardship team. Given that the study focused on uncomplicated BSIs, the treatment duration was optimized to be consistent with prior literature demonstrating 7 days from source control to be noninferior to longer durations in most cases [6, 10]. As such, a reduction of total mean antibiotic duration in the post-intervention period was expected. The significant reductions in total intravenous therapy and total duration of therapy are pragmatically significant as they relate to medication costs, nursing hours, and total healthcare expenditure [5, 11]. From a health system standpoint, this approach can decrease the higher cost of both inpatient and outpatient intravenous therapy.

We did not identify any concerning safety signals related to 30-day or 90-day mortality rates, readmission, adverse events, or composite clinical failure. However, this study was not powered to draw definitive conclusions regarding the clinical efficacy of oral versus intravenous therapy. Moreover, we did not collect the specific dosing regimen used for each patient; however, all recommended doses based on the guidance listed in Supplementary Table 2 were accepted by providers and adjusted according to the patient's renal function at the time of oral transition. Additional limitations include the single-center study design, conducted within a setting with an established and robust antimicrobial stewardship program, which may limit generalizability to institutions with fewer resources. The quasi-experimental design introduces the potential for temporal confounding due to time-related changes in practice. Notably, no changes were made to local practices for ID consultation, despite a higher rate of consultation in the post-intervention period. Moreover, the magnitude of improvement in oral antibiotic use observed after intervention makes it less likely that these changes alone account for the findings. Rates of ID consultation were higher and fewer patients had cardiac devices in the post-intervention group; however, our multivariable analysis suggests that these factors were not independently associated with oral transition (Supplementary Table 4). Finally, therapeutic drug monitoring was not performed for the oral antibiotics used, which may be a useful future direction to ensure optimal drug exposure and treatment efficacy.

While there is a growing body of evidence supporting the clinical efficacy of oral therapy in the treatment of non-staphylococcal BSIs, the implementation in routine clinical practice can lag behind. This study demonstrates that a real-time stewardship-driven intervention can significantly increase the rate of oral transition without compromising clinical or safety outcomes. The standardized oral transition criteria and oral antibiotic regimens used here offer a reproducible framework that may assist institutions aiming to adopt similar strategies, particularly those seeking to support clinicians with less experience in managing BSIs with oral agents. Our findings also reinforce the value of prospective audit and feedback interventions in optimizing antimicrobial use. As such, targeted stewardship interventions have the potential to improve quality of care, reduce healthcare utilization, and support broader adoption of evidence-based practices for the management of BSIs. Future work should evaluate the scalability of this model across diverse healthcare settings and explore additional tools, such as therapeutic drug monitoring, to further ensure appropriate oral antibiotic exposure.

## Supplementary Material

ofag120_Supplementary_Data

## Supplementary Data

Supplementary materials are available at *Open Forum Infectious Diseases* online. Consisting of data provided by the authors to benefit the reader, the posted materials are not copyedited and are the sole responsibility of the authors, so questions or comments should be addressed to the corresponding author.
