# Supplementary material for: Implementation of an Early Transition to Oral Antibiotics for Patients With Nonstaphylococcal Bacteremia via Real-Time Stewardship Intervention
Source: Open Forum Infect Dis. 2026 Mar 4;13(3):ofag120. doi: 10.1093/ofid/ofag120 (PMC13006138; doi:10.1093/ofid/ofag120)
Supplement: ofag120_Supplementary_Data [file ofag120_supplementary_data.docx]

**Supplementary Table 1. Standardized Criteria Used to Recommend Oral Antibiotic Transition**

1. **Bacteremia with appropriate pathogen and antimicrobial susceptibility results available**
   1. *Enterobacterales, Streptococcus spp., Enterococcus faecalis*
   2. At least 1 blood culture bottle positive. Must not be deemed a contaminant by treating physician
2. **Clinical stability defined as:**
   1. Off vasopressors >48 hours, afebrile x24 hours
   2. For patients with pneumonia associated bacteremia, oxygen requirement should be improving or stabilized
3. **Negative blood cultures for 48 hours if repeat blood cultures collected**
   1. No documented blood culture clearance required if no repeat blood cultures collected
4. **Source control achieved and no metastatic source of infection identified**
   1. Urinary tract − Exchange or removal of urinary catheter(s)/nephrostomy tube(s) − Removal of source of infection (e.g., stent, stone, obstruction) − No renal abscess​
   2. Intra-abdominal − Drainage of abscesses/collection − Debridement of infected necrotic tissues − Removal of infected mesh​
   3. ​Skin and soft tissue − Incision and drainage of abscess − Debridement of the infected/necrotic tissue or amputation​ if necessary
   4. ​Central venous catheter − Removal and exchange of vascular catheter​
   5. ​Unknown − No localizing signs or symptoms AND no source identified if imaging was performed​
   6. Must not have: Presence of associated endovascular infection such as endocarditis, bone and joint infection, central nervous system infection, complex intra-abdominal infections (lack of source control), imaging assurance [as needed]​
5. **No severe immunosuppression**
   1. Exclusions: Solid organ transplant < 1 year from transplant or <6 months from rejection treatment, bone marrow transplant < 1 year, ANC<1000, prednisone >20mg for >3 weeks, CD4<200
6. **Tolerating enteral medications, no malabsorption**
   1. Ensure patient is taking other enteral medications
   2. No short bowel, TPN dependence, or other mitigating factors

**Supplementary Table 2. Preferred Oral Antibiotic Regimens Recommended by Stewardship Team**

| **Organisms​** | **Preferred Oral treatment** | **Alternative Oral Treatment (based on AST results, allergies)** |
| --- | --- | --- |
| **All *Enterobacterales* (except those listed below)** | **-Amoxicillin:** 1000 mg PO q6h - q8h  **-Trimethoprim-sulfamethoxazole**: 8-12 mg/kg/day (trimethoprim component) PO divided q8- 12h (maximum dose of 960 mg trimethoprim component per day)​ | **-Ciprofloxacin**: 750 mg PO q12h​  **-Levofloxacin**: 750 mg PO q24h​  **-Amoxicillin-clavulanate:**875-125 mg PO TID |
| ***Citrobacter freundii, Enterobacter cloacae, Klebsiella aerogenes, Morganella morganii, Serratia spp*.​** | **-Trimethoprim-sulfamethoxazole**: 8-12 mg/kg/day (trimethoprim component) PO divided q8-12h (maximum dose of 960 mg trimethoprim component per day)​ | **-Ciprofloxacin**: 750 mg PO q12h​  **-Levofloxacin**: 750 mg PO q24h​ |
| ***Enterococcus faecalis*(not *E. faecium*)​** | **-Amoxicillin**: 1000 mg PO q6h ​- q8h | **-Linezolid**600mg PO q12h​ |
| ***Streptococcus spp*. (including viridans group, beta-hemolytic streptococci, *S. pneumoniae*)** | **-Amoxicillin**: 1000 mg PO q6h ​- q8h | **-Linezolid**600mg PO q12h​  **-Levofloxacin**: 750 mg PO q24h  **-Cephalexin**: 1000 mg PO q6h ​  **-Cefadroxil**†: 1000 mg PO q12h​  ​ |

†Robust PK studies have not been completed and benefits should outweigh risks before use of these agents. If penicillin allergic, avoid cephalexin and cefadroxil unless these agents specifically or other penicillin have been tolerated previously; patients were dosed based on recommendations and adjusted for renal function

**Supplementary Table 3. Blood Culture Pathogens**

| **Species** | **Pre-Stewardship Intervention** | | **Post-Stewardship Intervention** | |
| --- | --- | --- | --- | --- |
| **Gram-negative** | **N = 65** | **Percentage** | **N=55** | **Percentage** |
| **E. coli** | **26** | **31.7%** | **22** | **28.9%** |
| **K. pneumoniae** | **18** | **22.0%** | **14** | **18.4%** |
| **E. cloacae** | **8** | **9.8%** | **4** | **5.3%** |
| **Proteus mirabilis** | **3** | **3.7%** | **3** | **3.9%** |
| **Serratia marcescens** | **3** | **3.7%** | **2** | **2.6%** |
| **Klebsiella oxytoca/Raoultella** | **4** | **4.9%** | **1** | **1.3%** |
| **Citrobacter freundii** | **1** | **1.2%** | **3** | **3.9%** |
| **Morganella morganii** | **1** | **1.2%** | **1** | **1.3%** |
| **Providencia stuartii** |  |  | **1** | **1.3%** |
| **H. influenzae** |  |  | **1** | **1.3%** |
| **Citrobacter amalonaticus complex** |  |  | **1** | **1.3%** |
| **Pantoea septica** |  |  | **1** | **1.3%** |
| **Hafnia alvei** |  |  | **1** | **1.3%** |
| **Klebsiella variicola** | **1** | **1.2%** |  |  |
| **Gram-positive** | **N = 17** | **Percentage** | **N = 21** | **Percentage** |
| **Enterococcus faecalis** | **7** | **8.5%** | **3** | **3.9%** |
| **Group B Streptococci** | **4** | **4.9%** | **3** | **3.9%** |
| **Streptococcus salivarius group** | **2** | **2.4%** | **1** | **1.3%** |
| **Streptococcus mitis group** | **3** | **3.7%** | **1** | **1.3%** |
| **Streptococcus gallolyticus group** | **1** | **1.2%** |  |  |
| **S. dysgalactiae** |  |  | **2** | **2.6%** |
| **S. pyogenes** |  |  | **4** | **5.3%** |
| **S. anginosus** |  |  | **4** | **5.3%** |
| **Streptococcus species** |  |  | **1** | **1.3%** |
| **S. gordonii** |  |  | **1** | **1.3%** |
| **S. constellatus** |  |  | **1** | **1.3%** |

**Supplementary Table 4. Characteristics associated with patients who did or did not transition to oral antibiotics**

| **Characteristic** | **No Oral Transition**  **(n=39)** | **Oral Transition**  **(n=119)** | **Univariate**  **p-value** | **aOR**  **(95% CI)** | **Multivariate**  **p-value** |
| --- | --- | --- | --- | --- | --- |
| **Age, years** | 70 (58.5–80.5) | 64 (51–72) | 0.015 | 0.98 (0.93 – 1.02) | 0.276 |
| **Male sex** | 22 (56.4%) | 70 (58.8%) | 0.85 |  |  |
| **Gram-positive organism** | 12 (30.8%) | 26 (21.8%) | 0.28 |  |  |
| **Urinary source** | **7 (17.9%)** | **46 (38.7%)** | **0.019** | **4.23 (1.16 – 15.47)** | **0.029** |
| **ICU admission** | **31 (79.5%)** | **41 (34.5%)** | **<0.001** | **0.10 (0.03 – 0.34)** | **<0.001** |
| **Pitt bacteremia score** | 3 (2–6.5) | 2 (0–3) | <0.001 | 0.96 (0.77 – 1.18) | 0.673 |
| **Charlson comorbidity index** | 6 (4.5–9) | 5 (3–8) | 0.014 | 0.85 (0.70 – 1.02) | 0.083 |
| **IV drug use** | 4 (10.3%) | 11 (9.2%) | 1.00 |  |  |
| **Cardiac device present** | 7 (17.9%) | 8 (6.7%) | 0.056 | 0.87 (0.14 – 5.19) | 0.874 |
| **Infectious Diseases consult** | 23 (59.0%) | 70 (58.8%) | 1.00 |  |  |
| **Post-stewardship intervention** | **5 (12.8%)** | **71 (59.7%)** | **<0.001** | **17.23 (4.6 – 64.5)** | **<0.001** |

Data are presented as median (interquartile range) or n/N (%), as appropriate.

**Supplementary Table 5. Oral Antibiotics**

| Antibiotic | Pre-Stewardship Intervention |  | Post-Stewardship Intervention |  | p-value |
| --- | --- | --- | --- | --- | --- |
| Amoxicillin | 1 | 2.1% | 15 | 21.1% | **0.002** |
| TMP/SMX | 7 | 14.6% | 14 | 19.7% | 0.63 |
| Amox/Clav | 8 | 16.7% | 14 | 19.7% | 0.81 |
| 1st gen cephalosporin | 2 | 4.2% | 4 | 5.6% | 1 |
| 2nd gen cephalosporin | 10 | 20.8% | 5 | 7.0% | **0.05** |
| 3rd gen cephalosporin | 2 | 4.2% | 0 | 0.0% | 0.16 |
| Levofloxacin | 2 | 4.2% | 13 | 18.3% | **0.03** |
| Ciprofloxacin | 13 | 27.1% | 6 | 8.5% | **0.01** |
| Linezolid | 3 | 6.3% | 0 | 0.0% | 0.06 |

Abbreviations: TMP/SMX, Trimethoprim / Sulfamethoxazole; Amox/Clav, Amoxicillin / Clavulanic acid; gen, generation

**Supplementary Figure 1. Oral Antibiotics**


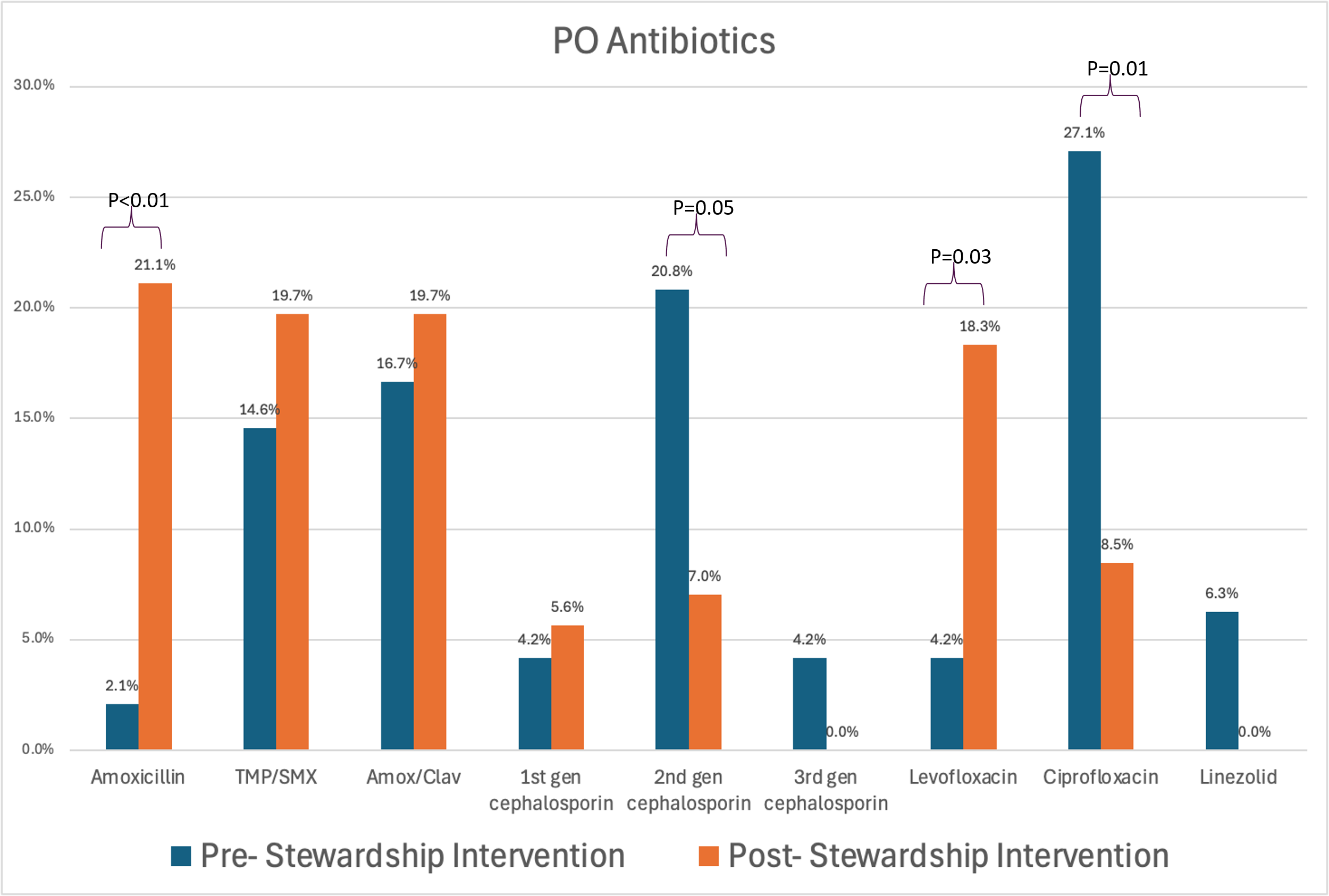


TMP/SMX, Trimethoprim / Sulfamethoxazole; Amox/Clav, Amoxicillin / Clavulanic acid; gen, generation
